# Beneficial effects of omega-3 fatty acid supplementation in schizophrenia: possible mechanisms

**DOI:** 10.1186/s12944-020-01337-0

**Published:** 2020-07-03

**Authors:** Mei-Chi Hsu, Yung-Sheng Huang, Wen-Chen Ouyang

**Affiliations:** 1 Department of Nursing, I-Shou University, No.8, Yida Road, Jiaosu Village Yanchao District, Kaohsiung, 82445 Taiwan; 2 College of Medicine, I-Shou University, No.8, Yida Road, Jiaosu Village Yanchao District, Kaohsiung, 82445 Taiwan; 3 Department of Geriatric Psychiatry, Jianan Psychiatric Center, Ministry of Health and Welfare, No.539, Yuzhong Rd., Rende Dist., Tainan City, 71742 Taiwan; 4 Department of Nursing, Shu-Zen Junior College of Medicine and Management, No.452, Huanqiu Rd. Luzhu Dist, Kaohsiung, 82144 Taiwan; 5 Department of Psychiatry, College of Medicine, Kaohsiung Medical University, No.100, Shin-Chuan 1st Road, Sanmin Dist., Kaohsiung, 80708 Taiwan

**Keywords:** Docosahexaenoic acid, positive and negative symptoms, cognitive functions, neurotransmission, prodromal phase, first-episode schizophrenia, chronic schizophrenia

## Abstract

**Background:**

Schizophrenia is a serious long-term psychotic disorder marked by positive and negative symptoms, severe behavioral problems and cognitive function deficits. The cause of this disorder is not completely clear, but is suggested to be multifactorial, involving both inherited and environmental factors. Since human brain regulates all behaviour, studies have focused on identifying changes in neurobiology and biochemistry of brain in schizophrenia. Brain is the most lipid rich organ (approximately 50% of brain dry weight). Total brain lipids is constituted of more than 60% of phospholipids, in which docosahexaenoic acid (DHA, 22:6n-3) is the most abundant (more than 40%) polyunsaturated fatty acid (PUFA) in brain membrane phospholipids. Results from numerous studies have shown significant decreases of PUFAs, in particular, DHA in peripheral blood (plasma and erythrocyte membranes) as well as brain of schizophrenia patients at different developmental phases of the disorder. PUFA deficiency has been associated to psychotic symptoms and cognitive deficits in schizophrenia. These findings have led to a number of clinical trials examining whether dietary omega-3 fatty acid supplementation could improve the course of illness in patients with schizophrenia. Results are inconsistent. Some report beneficial whereas others show not effective. The discrepancy can be attributed to the heterogeneity of patient population.

**Methods:**

In this review, results from recent experimental and clinical studies, which focus on illustrating the role of PUFAs in the development of schizophrenia were examined. The rationale why omega-3 supplementation was beneficial on symptoms (presented by subscales of the positive and negative symptom scale (PANSS), and cognitive functions in certain patients but not others was reviewed. The potential mechanisms underlying the beneficial effects were discussed.

**Results:**

Omega-3 fatty acid supplementation reduced the conversion rate to psychosis and improved both positive and negative symptoms and global functions in adolescents at ultra-high risk for psychosis. Omega-3 fatty acid supplementation could also improve negative symptoms and global functions in the first-episode patients with schizophrenia, but improve mainly total or general PANSS subscales in chronic patients. Patients with low PUFA (particularly DHA) baseline in blood were more responsive to the omega-3 fatty acid intervention.

**Conclusion:**

Omega-3 supplementation is more effective in reducing psychotic symptom severity in young adults or adolescents in the prodromal phase of schizophrenia who have low omega-3 baseline. Omega-3 supplementation was more effective in patients with low PUFA baseline. It suggests that patients with predefined lipid levels might benefit from lipid treatments, but more controlled clinical trials are warranted.

## Introduction

Schizophrenia, a serious long-term psychological disorder, affects about 1 percent of the population worldwide [1]. It is typified by positive symptoms (such as hallucinations and delusions), negative symptoms (including anhedonia, alogia, avolition, etc.), severe behavioral problems and cognitive function deficits (e.g., impaired psychological functioning) [2]. To date, the cause of schizophrenia is not fully understood. The heterogeneity of symptoms suggests the cause of schizophrenia is multifactorial, involving both genetic and environmental factors (e.g., prenatal infection, maternal malnutrition etc.) [3]. However, genes alone cannot cause schizophrenia as studies in identical twins show genetic factor represents only 50% of risk rates [4]. Nonetheless, those people with defective genes may be more vulnerable to various environmental risk factors and develop the disease [5].

Generally, the onset of schizophrenia begins during late adolescence or early adulthood [6], when the maturation of the brain and myelination is taking place. Disruption of normal brain development during prenatal or early postnatal period causes brain to be defective in function, suggesting that deleterious central nervous system (CNS) may play a pivotal role in development of this disease. Indeed, patients with schizophrenia in comparison with healthy controls, have a significant decrease in total brain, grey matter (GM), and white matter (WM) volumes and density, while a significant increase in lateral and third ventricle volumes [7, 8]. The structural change of brain is progressively developed before onset in the ultra-high risk (UHR) for psychosis subjects, during late adolescence or early adulthood, and continuous through the lifespan of the patients [9–14]. Postmortem studies in chronic schizophrenia have also shown brain abnormalities, which occur in specific areas like amygdala, basal ganglia, cerebellum, corpus callosum, inferior parietal lobule, medial temporal lobe, prefrontal cortical areas, superior temporal gyrus, and thalamus [15]. Since these abnormalities are not found in unaffected siblings and healthy controls, suggesting that the structural brain abnormalities are most likely related to the illness.

Since human brain controls all brain functions and behavior, schizophrenia is considered as a brain disorder. To better understand the cause of this disease, numerous studies have focused on identifying changes in neurobiology and biochemistry of brain in schizophrenia. Brain is the most lipid rich organ (approximately 50% of brain dry weight). Phospholipids constitute more than 60% of the total membrane lipids. Brain phospholipids contain two families of polyunsaturated fatty acids (PUFAs): omega-3 (or n-3) and omega-6 (or n-6). The most abundant omega-3 fatty acid is docosahexaenoic acid (DHA, 22:6n-3), followed by eicosapentaenoic acid (EPA, 20:5n-3), and docosapentaenoic acid (DPA, 22:5n-3), whereas the main omega-6 fatty acid is arachidonic acid (AA, 20:4n-6). DHA accounts for 40% of the total membrane phospholipids fatty acids in brain [16]. Thus, DHA is essential for the normal neurological development and plays a critical role in the maintenance of biological processes including receptor binding, neurotransmission, and signal transduction and cognitive functions such as learning and memory [17–19]. Therefore, the homeostasis of brain phospholipid and PUFAs in patients with schizophrenia is an important study subject for better understanding the relationship between the specific lipid molecules and structural and functional changes in brain. So that the strategy as how to deter the development and progress of this disease can be developed. Early, Horrobin [20] has proposed the Phospholipid Hypothesis of Schizophrenia. According to this hypothesis, an elevated phospholipase A2 (PLA2) activity in patients, which releases PUFAs, mainly DHA and AA, from membrane phospholipids has caused PUFA deficiency, and a progressive degradation of brain tissues. This produces aberrant neurotransmission, psychological symptoms, and impairment of cognitive and brain functions.

Indeed, ample evidence has shown significant reduction of PUFAs, in particular AA and DHA in peripheral blood (plasma and erythrocyte membranes) of schizophrenia patients at different development stages (including ultra-high risk individuals, un-medicated first-episode and chronic patients [21–33]. Two meta-analyses have also confirmed significant reduction of AA, and DHA, in medication-free schizophrenia patients, and patients treated with antipsychotics [30, 34]. There are also studies showing no differences or even increases of AA and DHA levels in patients with schizophrenia as compared to healthy subjects (references). A study conducted by Medema et al. [35] has reported increased erythrocyte DHA, DPA and AA in a large cohort of schizophrenia patients and unaffected siblings compared to controls. Discrepancy in findings between Medema et al. [35] and 2 meta-analyses could be due to different measurement units used in presenting fatty acid content. Medema et al. [35] reported fatty acid content by absolute concentration (picomole/10^6^ erythrocytes), whereas studies included in meta-analyses and others by percentages. The significant increases in DHA and AA reported in the study by Medema et al. [35] were lost when fatty acids were presented as percentages. Another difference could be due to heterogeneity of patient populations. 61.9% of patients in Medema et al. (2016) received atypical antipsychotic medication, which is known to increase the biosynthesis of PUFAs and raise the levels of PUFAs [36].

Reports have also shown significant breakdowns of phospholipids and reduction of DHA in brain orbitofrontal cortex (Brodmann area 10, BA10), and suggested that DHA deficit in brain is associated with the pathogenesis of schizophrenia [37–39]. However, there are reports showing no difference of DHA levels in other brain regions (amygdala, prefrontal cortex) between schizophrenic patients and controls, suggesting abnormalities of PUFA levels are region-specific [40, 41]. Since these abnormalities were not observed in unaffected siblings and healthy controls, the structural brain abnormalities found in patients are most likely related to the illness itself [8, 12].

In this review, the cause of brain PUFA deficit in patients, the role of PUFAs in the development of this disorder, and beneficial effects of omega-3 supplementation on symptoms and cognitive functions were examined, and the potential mechanisms underlying these beneficial effects discussed.

## Methods

The main aims of this review are twofold. First, the role of omega-3 PUFAs in the development of schizophrenia was addressed, and preclinical and clinical evidence regarding the beneficial effect of omega-3 supplementation on symptoms and cognitive functions reviewed. Secondly, the potential mechanisms underlying the beneficial role of omega-3 PUFAs on schizophrenia were discussed.

To achieve these aims, a comprehensive literature search in electronic databases, such as PubMED, EMBASE and PsycINFO was conducted. The following terms: omega 3 fatty acids, cognition, symptoms, and schizophrenia were used for the search. The inclusion criteria were: studies contained original data on effects of omega-3 PUFAs in symptoms, functions and cognition in schizophrenia and published in English between 2000 and 2020.

### Data extraction and quality assessment

The quality of all eligible studies and outcomes were carefully evaluated. All authors independently extracted each of the selected studies and evaluated the study quality. The followings: primary aim, attributes, context and exemplar of omega-3 PUFA, evaluation or description of omega-3 PUFA formulas, outcomes, and possible bias were checked and analyzed inductively. Data from selected studies with good quality in term of methods, outcome measures, and statistics analysis were extracted, and whether these selected studies exhibited any major limitation that could negatively impact or influence the interpretation of the study findings evaluated.

## Results

### Prevalence and causes of polyunsaturated fatty acid deficiency in schizophrenia

**Fig. 1:**
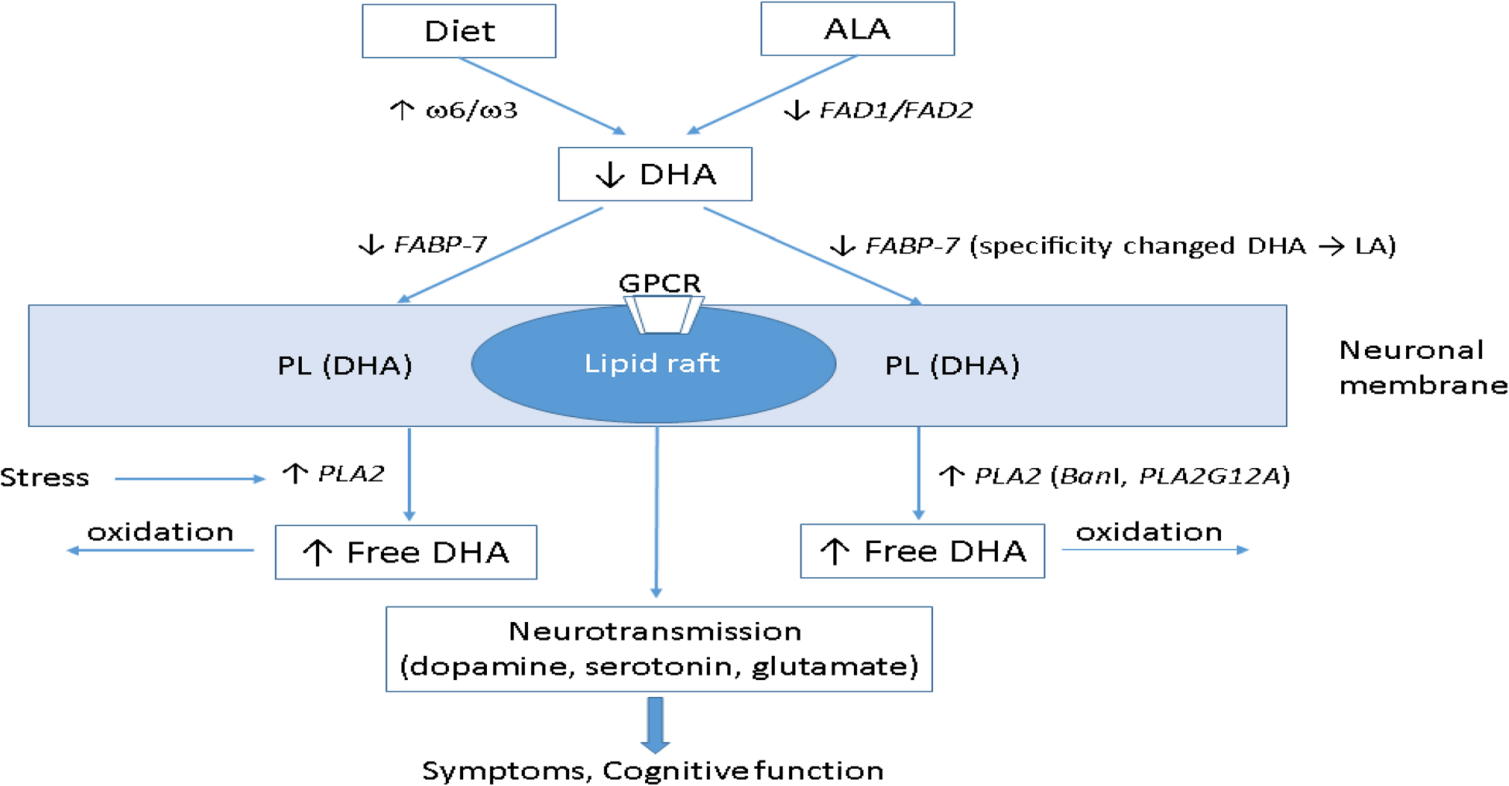
Causes of PUFA (DHA in particular) deficiency in schizophrenia. Cause may be due to high ω6/ω3 diet, low synthesis due to abnormal metabolic enzymes, or low absorption due to mutated fatty acid binding protein; and elevated phospholipase A2 activity which release PUFAs from cell membrane. Abbreviations: AA, arachidonic acid (20:4n-6); ALA, alpha-linolenic acid (18:3n-3); DHA, docosahexaenoic acid (22:6n-3); FABP-7, fatty acid binding protein; FAD1/FAD2, delta-5 and delta-6 fatty acid desaturases; GPCR, G-protein coupling receptor; LA, linoleic acid (18:2n-6); PL, phospholipids; PLA2, phospholipase A2

Ample evidence has shown that PUFA deficiency occurs in schizophrenia, which may be caused by many factors. A simple scheme (Figure 1) outlines the possible factors involved in the process of PUFA deficiency.

### Reduced synthesis and uptake of long-chain PUFAs

During brain development, brain possesses the enzymes required for the synthesis of DHA and AA from alpha-linolenic acid (ALA, 18:3n-3) via EPA, and linoleic (LA, 18:2n-6) acid, respectively. The concentrations of DHA and AA increase sharply. Inadequate brain accumulation of DHA during this period can result in an omega-3 PUFA-deficiency which impair the cortical structure and functional maturation [42], and increase the risk for schizophrenia [43]. In adult brain, the synthesis rate decreases significantly [32, 42]. In schizophrenia, genetic variation, such as fatty acid desaturase (FAD), FAD1/FAD2 genes, has further reduced the ability to synthesize long-chain PUFAs [44, 45]. Normally, the consumption rate of AA and DHA by adult human brain was estimated to be 17.8 and 4.6 mg/day, respectively [46]. To maintain normal structure and function, brain relies on a constant supply of AA and DHA from the food via blood [47]. Unfortunately, schizophrenia patients often consume unbalanced diet (high omega-6:omega-3 ratio). Pawełczyk et al. [48] have reported that UHR individuals consumed significantly higher proportion of omega-6 fatty acids (LA and AA) whereas less of omega-3 fatty acids (ALA, EPA, and DHA) in comparison with individuals who did not develop psychosis. Similarly, patients with chronic schizophrenia also have a poor diet (high intake of saturated fat and low polyunsaturated fat) [49–51].

### Abnormal fatty acid binding protein in schizophrenia

PUFA depletion could be caused by abnormal fatty acid binding proteins (FABPs) in the brain of schizophrenia. FABPs, the intracellular lipid trafficking proteins, play essential roles in transporting fatty acids into the cytoplasm and appropriate intracellular compartments. In human, there are 3 FABPs family members (FABP3, FABP5 and FABP7) found in mature neurons, neural progenitor cells and neural stem/progenitor cells in brain [52, 53]. Each shows different fatty acid preference. FABP3 binds preferentially to omega-6 PUFAs (e.g., AA) [54]. FABP5 favors saturated (e.g., stearic acid), and monounsaturated fatty acids (e.g., oleic acid) [52, 54]. Evidence has shown exclusively in schizophrenia that two genetic variations of FABP7 (FABP7 S86G and FABP7 V126L) change preference from DHA to LA [55]. This abnormality would result in an unbalanced DHA mobilization and utilization, and a greater reduction of DHA relative to omega-6 PUFAs in brain cell membrane [29].

### Elevated phospholipase A2 activity in schizophrenia

Elevated PLA2 activity has been suggested in the lipid membrane hypothesis as the cause of PUFA depletion in schizophrenia. PLA2 is an enzyme that hydrolyzes fatty acid in position 2 (sn-2) from the membrane phospholipids, producing a free fatty acid and a 2-lysophospholipid [56]. In brain, there are three major PLA2 enzymes: a calcium-dependent AA-specific cytosolic PLA2 (cPLA2); a calcium-dependent AA-specific secretory PLA2 (sPLA2); and a calcium-independent DHA-specific PLA2 (iPLA2) [56–59]. Smesny et al. [60] have shown PLA2 activity increased in UHR individuals and patients with first episode. Post-mortem brain studies have shown that increased iPLA2 activity is associated with structural brain degradation in the first episode schizophrenia patients [61, 62]. An increased DHA-specific iPLA2 activity in the brain of patients enhances the release of DHA from the DHA-containing phospholipids, change the physicochemical properties (e.g., fluidity, permeability) of synaptic membranes, and result in an abnormal neurotransmission in the brain of schizophrenic patients [63]. Šakić et al. [64] have suggested an association between iPLA2 activities and the length of illness and frequency of episodes occurred. The cause of increased PLA2 activity in brain in patients with schizophrenia is not clear, but increased levels of stress-induced cytokines in schizophrenia may stimulate the activity [65–67]. The increased PLA2 activity could also be caused by variants of genes expressing the PLA2. Increases in *Ban* I polymorphism of the cPLA2 gene and *PLA2G12A* polymorphism of the sPLA2 gene have been shown in schizophrenia of different ethnic groups [68–71]. Both cPLA2 and sPLA2 catalyze the release of arachidonic acid from membrane phospholipids for production of inflammatory eicosanoids.

### Increased Oxidative Stress in Schizophrenia

PUFA depletion in schizophrenia patients could be due to an increase in oxidative stress [72]. In adult human, brain accounts for approximately 20% of total body oxygen consumption even though it comprises only 2% of the body weight. Maintaining normal oxidative stress requires adequate antioxidant capacity, which is relatively low in brain as compared to other tissues. Therefore, brain is vulnerable to oxidative stress. Studies have shown in schizophrenia an increase in oxidative stress, in conjunction with a decrease in antioxidant defense enzymes **(**e.g., superoxide dismutase (SOD), catalase, and glutathione peroxidase) in schizophrenia [73–81]. The unbalance in pro- and anitioxidants may have increased susceptibility of brain PUFAs to oxidative damage and subsequently contributed to the deterioration of brain structure and cognitive impairment during the course of the disease [23, 24, 76, 82–94]. The DHA-rich region, such as **PFC**, is the most prone to oxidation damage [95].

### Association between PUFA deficiency and symptoms/cognition in schizophrenia

Evidence has shown that low PUFA levels are associated with negative and positive symptoms [24, 96–100] in patients with schizophrenia. Studies also show the blood levels of PUFAs, particularly DHA, are negatively correlated with the severity of symptoms [98, 101, 102].

Cognitive functioning refers to many different mental abilities including attention, memory, language, attention, perception, problem solving, decision making, etc. [103]. Cognitive deficits, especially in memory abilities are found in about 75–85% of schizophrenia patients [104]. It impacts negatively on psychosocial functioning in schizophrenia [105]. Generally, cognitive deficits are found early in UHR individuals and at the onset of illness [106, 107], become evident in first-episode, treatment-naïve patients [2], and continue to decline as illness progressed [108]. Thus, cognitive symptoms may serve as a prognostic marker and predictor of schizophrenia [109]. Several studies have shown that abnormality in PUFA (mainly DHA) levels in UHR and schizophrenic patients is associated with memory, language and cognitive impairments [100, 110–114]. PUFAs, particularly DHA, play an important role in maintaining brain function and neural transmission [115–117].

### Effect of omega-3 fatty acid supplementation on symptoms and cognitive function in schizophrenia

The fact that significant reduction of omega-3 PUFA levels is seen in plasma, red blood cells (RBC) and brain in patients with schizophrenia, has led to a number of open-label and randomized clinical trials examining whether dietary supplementation with omega-3 PUFAs could improve the course of illness in patients with schizophrenia.

However, results from many studies examining effects of omega-3 supplementation on symptoms in schizophrenia were inconsistent. Some show reduced conversion rate to psychosis in UHR individuals [118–120], incidence rate, improved prognoses with greater efficacy over placebo in first-episode [48, 121–124] and chronic patients [24, 125–128], while others showed no differences between schizophrenia and control groups [129, 130]. One study [131] reported worse in symptoms. Several meta-analyses [132, 133] and an early review of these clinical trials [134] failed to make plausible conclusions with respect to the therapeutic benefit of omega-3 PUFA supplements in this disease. However, a very recent review has shown favorable impacts of dietary supplementation of omega-3 fatty acids as a therapeutic option in mental disorder [135].

Fenton et al. [136] have carried out a randomized-controlled trial (RCT) investigating the add-on effects of EPA (3 g/d) on cognitive performance in antipsychotic treated patients with schizophrenia [136]. After 16-week trial, the authors found no difference in test scores of residual symptoms or cognitive performance between participants received EPA and patients randomized to placebo [136]. On the other hand, studies have shown that dietary supplementation with DHA improves memory and cognitive functions in healthy elderly subjects [137–140] and in patients with mild cognitive impairment [141]. One possible mechanism underlying the improved cognitive performance is related to the improved DHA status and behavioral development [142].

### Rationale for discrepancy in findings

The discrepancy of findings from different studies, could be due to heterogeneity of patient population, for example, different developmental stages. When omega-3 PUFAs were supplemented to UHR adolescents for a period of 12 weeks, Amminger et al. [118–120] found a significant reduction of the rate of conversion to first-episode schizophrenia, and the beneficial effects continued for a long period (6.7 years). They found that red blood cell PUFA level were lower in UHR as compared to normal [33]. However, in a large international trial, McGorry et al. [143] failed to observe effectiveness in preventing the conversion into first-episode. The authors attributed the lack efficacy of omega-3 treatment to the fact that all patients in both treated and placebo groups received normal healthy diets during the study. Indeed, Amminger and colleagues [144] have recently reported that the failure to show benefits of omega-3 fatty acid supplementation in UHR adolescents as compared to placebo by McGorry et al. [141] was due to the presence of omega-3 fatty acids in the diet and the body tissue of participants in the placebo group. Nonetheless, a placebo-controlled RCT by Pawełczyk et al. [48] comparing the efficacy of intervention with omega-3 fatty acids as an added on to antipsychotic medication, found that omega-3 fatty acids could significantly reduce the severity of symptoms and rate of relapse in first-episode schizophrenia. Since Pawełczyk and colleagues [145] have found that the subjects in the UHR group and the first episode schizophrenia patients consumed significantly higher level of omega-6 fatty acids and less of omega-3 fatty acids in comparison with healthy controls. Amminger et al. [144] have shown that increases of omega-3 levels predict improvement in symptoms and functioning in youth at UHR for psychosis. Taken together, it is possible that the efficiency of omega-3 intervention was due in part to the presence of omega-3 deficiency in many of those participants prior to treatment. Thus, omega-3 PUFA supplementation may not be beneficial for individuals who already have high omega-3 fatty acid levels at baseline.

### Effects of omega-3 fatty acid supplementation on brain structure and functions:

#### Reduce degradation of brain

Phospholipid breakdown and omega-3 PUFA deficit is known due to a pathological increase in PLA2 activity observed in brain of schizophrenia. Omega-3 supplementation has significantly reduced the intracellular PLA2 activity [146]. More specifically, EPA has been shown to inhibit PLA2 activity reducing the degradation of brain and thus, exert some effects in the treatment of schizophrenia. Administration of omega-3 fatty acids (mainly EPA), can inhibit PLA2 activity reducing the degradation of brain structure in schizophrenia [146].

#### Replenish brain DHA content

The beneficial action of omega-3 PUFA may act through improvement in biochemical and physical properties of brain cell membranes [72, 147–149]. DHA is the major omega-3 fatty acid found in nerve cell membrane phospholipids in brain cortical grey matter. DHA constitutes about 15% of total fatty acids in the adult human prefrontal cortex (PFC) [37, 42, 150]. Other omega- PUFAs, such as EPA and DPA, comprise less than 1% of total brain fatty acid composition [151]. Evidence has shown lower level of DHA in brain in schizophrenia patients [36, 39]. Such region-specific changes in brain phospholipid metabolism and fatty acid composition may affect physicochemical properties such as fluidity and permeability of neuronal cell membrane, which in turn, modulate the activities of membrane bound enzymes and neurotransmission system (such as receptors) located on the membrane (lipid rafts). DHA supplementation can replenish the membrane DHA content.

#### Reduce oxidative stress in schizophrenia

The possible mechanism underlying the beneficial action of omega-3 PUFAs may be via enhancing the anti-oxidative intracellular defense system [152]. Three intervention studies have reported the effect of omega-3 PUFA supplementation on levels of oxidative stress markers [123, 153, 154]. Sivrioglu et al. [153] studied the effect of a 4-month intervention with a combination of omega-3 PUFAs and antioxidants (vitamin E and C) on total antioxidant capacity (TAC) in medicated chronic schizophrenia patients. They found that the treatment significantly reduced the severity of positive and negative symptoms, levels of RBC-SOD. As an increase in SOD was a compensatory response to the increased production of ROS in schizophrenia patients, a reduction in levels of RBC-SOD indicates that intervention with a combination of omega-3 PUFAs and antioxidants can reduce the oxidative stress.

Smesny et al. [154] examined the data from the intervention study reported by Amminger et al. [119]. They assesses the effect of a combination of omega-3 PUFAs and vitamin E supplementation on tocopherol and glutathione (GSH) levels in erythrocyte membrane in individuals at high clinical risk. They found that intervention significantly increased RBC tocopherol, but reduced total RBC-GSH level. The authors conclude that supplementation with omega-3 PUFAs seems to support the antioxidant capacity at membrane level resulting in a decreased need for GSH. The authors suggested that inclusion of antioxidants (vitamin E and GSH) may account for the effectiveness of omega-3 PUFA supplementation in high clinical risk individuals.

Pawelczyk et al. [123] conducted a 6-month placebo-controlled RCT composed of 2.2 g/day of omega-3 PUFAs in first episode schizophrenia. The authors assessed whether the clinical effectiveness of omega-3 PUFAs were associated to changes in oxidative stress indices, and found a significant reduction of 8-isoprostane F_2α_ level, an oxidative stress index, and an increase in plasma TAC in patients. All these results from studies carried out in different developmental stages (UHR, first-episode or chronic schizophrenia), seem to be consistent that supplementation with omega-3 PUFAs can alleviate oxidative stress.

#### Modulation of neuro-inflammation in schizophrenia

Another possible mechanism underlying the beneficial action of omega-3 PUFAs may act through modulation of the inflammatory responses [155, 156]. Evidence has indicated that chronic neuro-inflammation in brain is one of the risk factors in the pathophysiology of schizophrenia [155–159]. Neuro-inflammation is distinguished by the activation of microglial cells [160]. The activated microglia increases the production and release of pro-inflammatory cytokines [161, 162], and subsequently, the formation of pro-inflammatory prostaglandin E2 (PGE2).

It has been shown that pro-inflammatory cytokines were increased in both serum and cerebrospinal fluid (CSF) in first-episode schizophrenia [163–165], and patients with chronic schizophrenia [166]. Postmortem studies have also shown inflammatory markers in the dorsolateral prefrontal cortex, and microglial activity and microglial cellular density were all increased in schizophrenic patients [165, 167–169]. The pro-inflammatory cytokines increase PLA2 activity and breakdown of membrane phospholipids in schizophrenic patients [38, 170].

Numerous animal and clinical studies have indicated that omega-3 fatty acids have anti-inflammatory properties and inflammation resolving effects. The anti-inflammatory effect of omega-3 fatty acids is moderated by competing (mainly EPA) with AA for incorporation into cell membrane phospholipids, and interfering with conversion of AA to form inflammatory eicosanoids, PGE2. Smesny et al. [145] observed that omega-3 fatty acid supplementation decreased significantly the intracellular PLA2 activity in young adults at UHR for psychosis. Puri et al. [171] found that EPA supplementation increased cerebral phospholipid biosynthesis whereas decreased phospholipid breakdown by inhibiting PGE2-induced PLA2 activity. This results in reduced neuronal phospholipid turnover and neuro-inflammation, whereas normalized cerebral phospholipid metabolism. In addition, DHA and EPA are precursors for the potent anti-inflammatory mediators, such as resolvins and neuroprotection Ds, which can actively limit inflammation and promote resolution [63, 172–174]. Thus, through inhibition on formation of inflammatory eicosanoids, and formation of anti-inflammatory mediators, omega-3 fatty acids exert the beneficial effects on schizophrenia. This mechanism may explain the beneficial effects of omega-3 fatty acids supplementation on schizophrenia by reducing the neuro-inflammation.

### Beneficial effects of omega-3 fatty acid supplementation: possible mechanisms

As shown by some but not all clinical intervention, omega-3 PUFA supplementation can be effective in alleviating symptoms and improving cognitive functions in patients with schizophrenia. The mechanism underlying these benefits is not clear. To facilitate the discussion, the present review focused on 13 clinical trials that showed positive response to the intervention (Table 1).

**Table 1:** Effects of omega-3 PUFA supplementation on symptoms and functions in schizophrenia

Trial	Authors	Omega-3 treatment			Effects of omega-3 treatment					ARR	95% CI	RRR
		EPA, DHA (mg/d)	Intervention (Follow-up)	ω3 level	Symptoms (PANSS scores)				Functions			
					T	P	N	G	GAF			
**Prodromal**												
1	Amminger et al. (2007) [118]	**EPA (**800**) + DHA (**700**)**	12 weeks	-	-	**↓**	-	**↓**	**↑**	18.5%	4.6-32.4	87.7%
2	Amminger et al. (2010) [119]	**EPA (**700**) + DHA (**400**)**	12 weeks (40 weeks)	-	**↓**	**↓**	**↓**	**↓**	**↑**	22.6%	4.8-40.4	82.2%
	Amminger et al. (2015) [120]	**EPA (**700**) + DHA (**400**)**	12 weeks (6.7 years)	-	**↓**	**↓**	**↓**	**↓**	**↑**	30.2%	10.1-50.4	75%
**First-episode psychosis**												
1	Berger et al. (2008) [121]	**EPA (**2000**)**	12 weeks	-	-	-	**↓**	-	**↑**			
	Wood et al. (2010) [122]	**EPA (**2000**)**	12 weeks	-	-	-	**↓**	-	-			
2	Pawełczyk et al. (2016) [48]	**EPA (**1320**) + DHA (**880**)**	26 weeks	-	**↓**	**NS**	**NS**	**↓**	**↑**			
	Pawełczyk et al. (2017) [123]	**EPA (**1320**) + DHA (**880**)**	26 weeks	-	-	-	**↓**	**↓**	**↑**			
3	Robinson et al. (2019) [124]	**EPA (**740**) + DHA (**400**)**	16 weeks	-	-	-	**↓**	-	-			
**Chronic schizophrenia**												
1	Peet et al. (2001) [125]	**EPA or DHA (**2000**)**	12 weeks	**↑**	**↓**	**↓**	**NS**	-	-			
2	Emsley et al. (2002) [126]	**EPA (**3000**)**	12 weeks	-	**↓**	-	-	-	-			
3	Arvindakshan et al. (2003) [24]	**EPA (**180**) + DHA (**120**)**	16 weeks	**↑**	**↓**	-	-	**↓**	-			
4	Sivrioglu et al. (2007) [127]	**EPA (**180**) + DHA (**120**)**	16 weeks	-	-	-	**↓**	-	-			
5	Jamilian et al. (2014) [128]	**EPA (**180**) + DHA (**120**)**	8 weeks	-	**↓**	**NS**	**NS**	**↓**	**-**			

Abbreviations: *95% CI* 95% Confidence Interval, *ARR* Absolute risk reduction, *DHA* docosahexaenoic acid (22:6ω3), *EPA* eicosapentaenoic acid (20:5n-3), *G* global subscale score, *GAF* global assessment of functioning scale, *N* negative subscale score, *NS* no significant difference, *P* Positive subscale score, *PANSS* Positive and Negative Syndrome Scale, *RRR* Relative risk reduction, *T* total subscale score, - information not available, ↓ decrease, ↑ increase

Two studies and one long-term follow-up from the same research group have shown that omega-3 supplementation improved both positive and negative symptoms and functions in UHR subjects [118–120]. Two studies supplemented the first-episode schizophrenia patients with EPA alone [121, 122], while 3 studies treated patients with a combination of EPA and DHA. All five studies show improvement in negative symptoms and functions. There are five studies examined the effect of EPA or DHA alone or a combination of both EPA and DHA [24, 125–128]. The improvement was found mainly on total positive and negative symptom scale (PANSS) subscale scores, but none in functions. In all these studies, omega-3 supplementation has raised the blood omega-3 fatty acid levels, which has been suggested as an indicator of PUFA levels in brain [42].

**Fig. 2:**
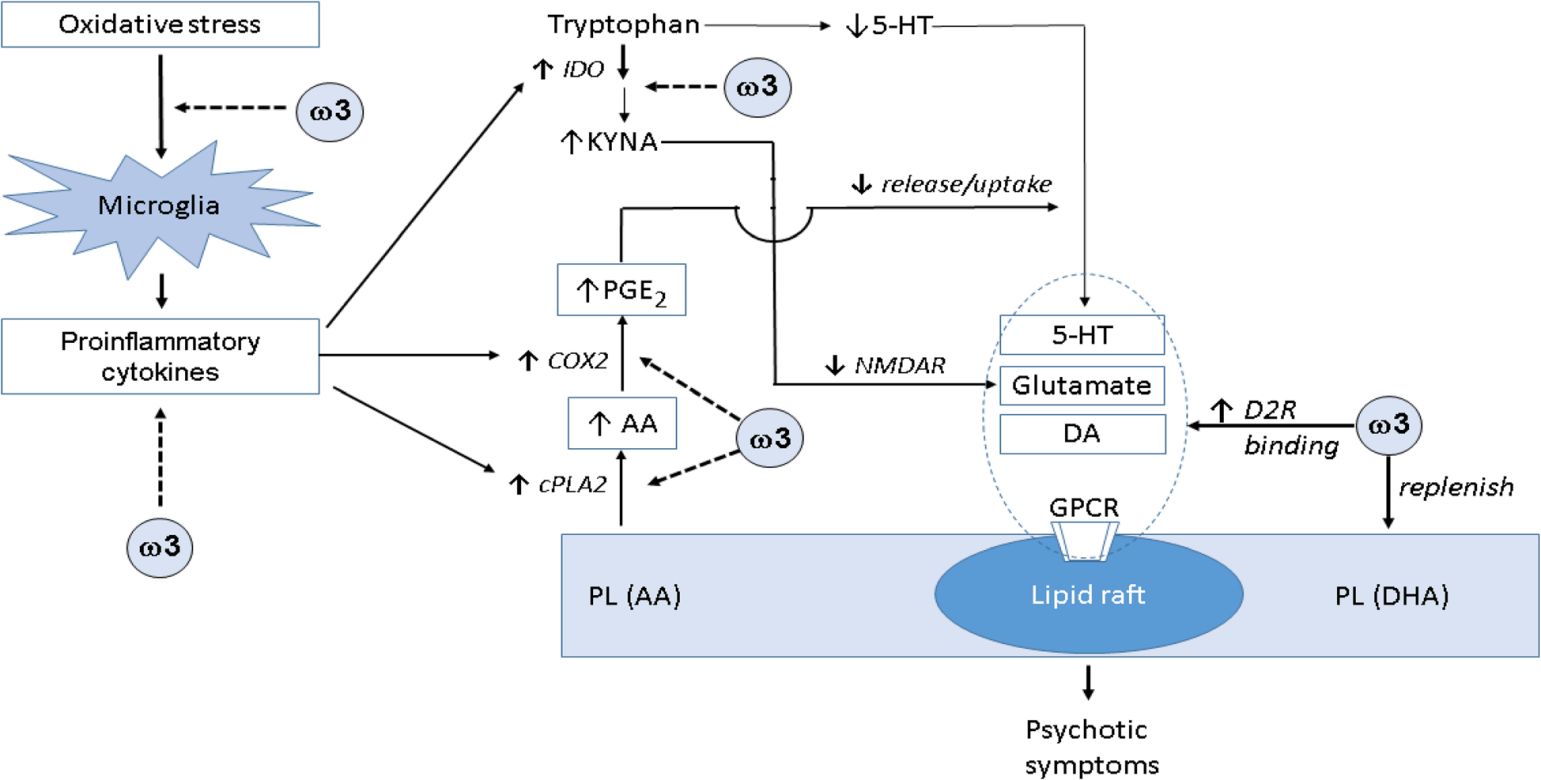
A scheme outlines mechanisms as how omega-3 fatty acids exert the beneficial effect on neurotransmission. Omega-3 fatty acids decrease oxidative stress; suppress formation of pro-inflammatory cytokines; inhibit production of KYNA, an antagonist of NMDA receptor, which increases glutamine levels; enhance release and uptake of serotonin, and facilitate dopamine binding to D2R by modulating membrane flexibility and permeability. Abbreviations: AA, arachidonic acid (20:4n-6); COX2, cyclooxygenase-2; DA, dopamine; DHA, docosahexaenoic acid (22:6n-3); D2R, dopamine receptor; 5-HT, serotonin, 5-hydroxytryptamine; KYNA, kynurenic acid; PGE2, prostaglandin E2

Abnormal symptoms and functions in schizophrenia are resulted of dysfunctional neurotransmission pathways. Thus, the beneficial effects of omega-3 supplementation may act through improving neurotransmission in patients (Figure 2).

#### Improve neuronal cell membrane, lipid rafts and G-protein-coupled receptor (GPCR) functions

Several hypotheses have attributed the abnormal neurotransmission systems including dopamine, glutamate, and serotonin to the cause of the symptoms of schizophrenia [175]. The recent dopamine hypothesis has proposed that transmission of dopamine (DA, 3,4-dihydroxyphenethylamine), a major neurotransmitter that transports signals between nerve cell endings in the brain, is abnormal in schizophrenia patients. Dopamine is produced and secreted by neuron mainly in the substantia nigra and ventral tegmental area (VTA) in midbrain. According to the hypothesis, the dopamine transmission from VTA to mesolimbic areas via the mesolimbic pathway is hyperactive, which is responsible for positive symptoms [176]. Whereas, dopamine transmission from VTA to the cortex (including PFC) and amygdala via the mesocortical pathway is hypoactive, which causes negative symptoms [176]. Since dopamine activities in PFC neurons are known to modulate the dopamine activities in mesolimbic area [177], a reduced dopamine activity in PFC further enhance activity in the limbic dopamine system. Levels of dopamine released in different regions are correlated to symptom severity [178]. Normal neuronal communication depends on the release of neurotransmitters from presynaptic vesicles into the synaptic cleft, and the uptake of GPCRs on the postsynaptic membrane [179]. These GPCRs and signaling proteins locate in lipid rafts in the brain neuronal membrane [177–179]. Increasing evidence indicates that lipid homeostasis in the nervous system changed during development in schizophrenia. Part of these changes can be attributed to altered fatty acid composition in lipid rafts. Generally, PUFA content as well as ratio of omega-6 and omega-3 fatty acids are important factors affecting the neuronal membrane integrity (e.g., plasticity and fluidity) [51]. Thus, alteration in membrane structure affects the function of membrane-bound proteins, availability of cell signalling molecules, and the behaviour of neurotransmitter systems and their physicochemical properties. These alterations then affect the GPCR activity located on lipid rafts and ultimately the neurotransmission [180]. Incorporation of highly unsaturated omega-3 PUFAs into neuronal membranes increases membrane fluidity, and modifies lipid raft organization [181–184], enhances affinity of receptors and facilitates receptor binding, and consequently, improves neurotransmission and signaling [185]. Stillwell et al. [184] have reported that DHA incorporation into brain membrane phospholipids affect cell signalling by altering lipid rafts.

Serotonin (5-hydroxytryptamine, 5-HT), another neurotransmitter, has also been suggested to play an important role in etiology and pathophysiology of schizophrenia. Eggers [185] has proposed that the dorsal raphe nucleus, the largest serotonergic nucleus in brain, is upregulated in response to stress or longterm stimulation in schizophrenia. This change aberrantly intensified serotonergic drive in the cerebral cortex, an early cause of the psychiatric features of the disease. Impairments in central 5-HT neurotransmission, which reflect the metabolism and turnover of serotonin in brain have been associated with behavioural and physiological abnormalities (violence, hostility, impulsivity and aggression), and psychiatric disorders (including schizophrenia). Patrick and Ames [186] have proposed the mechanism explaining how omega-3 fatty acids enhance serotonin function. They suggested that EPA in the brain inhibits the formation of PGE2 [187], which is known to inhibit the release of serotonin [188]. Thus, EPA facilitates the release of serotonin from presynaptic neurons. On the other hand, DHA increases the cell membrane fluidity and consequently, allows the binding of serotonin to the serotonin receptor in the postsynaptic neuron.

Glutamate, another major excitatory neurotransmitter, plays a dominant role in fast neurotransmission in human central nervous system. Evidence indicates that a lack of glutamatergic neurotransmission, is a key mechanism in the pathophysiology of schizophrenia [189, 190]. Hypofunction of glutamatergic signaling is mediated via abnormal N-methyl-D-aspartate receptor (NMDAR) which prevents glutamate from binding to the receptor, resulting in increasing levels of the excitotoxic glutamate. This may have contributed to the pathophysiology (e.g., morphological and structural brain changes), symptoms and cognitive deficits in the schizophrenia [191–195]. The hypofunction of NMDAR could be due to increased production of kynurenic acid (KYNA), which is antagonist of NMDAR. In schizophrenia, formation of serotonin from tryptophan was significantly reduced, due to increased conversion of tryptophan to KYNA. Omega-3 fatty acids suppress the formation of KYNA.

## Discussion

The cause of schizophrenia remains to be elusive. Evidence seems to suggest that cause of schizophrenia is multifactorial, occurrence of schizophrenia represents the cumulative effect of multiple factors (genetic or environmental). Abnormal PUFA metabolism may be one of the many factors involve in the development of this disorder. These factors have affected normal PUFA uptake and incorporation in nerve cells during brain development. Levels of PUFAs, particularly omega-3 fatty acids, i.e., EPA and DHA have been shown decreased in many schizophrenic patients.

### Omega-3 PUFA deficit causes structural and functional abnormalities in brain

Depletion of omega-3 PUFAs in patients with schizophrenia could be due to a long consumption of unbalanced high omega-6 diet during early developmental stages, and throughout the illness, abnormal uptake and transport of omega-3 fatty acids within neuronal cells, and increased release and oxidation of omega-3 fatty acids from the neuronal cell membrane phospholipids due to an elevated PLA2 activity.

PUFA deficit has resulted in many adverse effects seen in schizophrenia, such as abnormal brain structure, symptoms, aberrant neurotransmission and neuro-inflammation etc. Dysregulation of PUFA (including AA and DHA) metabolism at the early stage, could affect normal neural development, magnify inflammatory responses, and lead to aberrant neurotransmission. Omega-3 deficiency causes abnormal brain structure (lipid rafts), and subsequently the dysfunction of neurotransmitter receptors located on the surface (lipid rafts) of cell membranes, aberrant neurotransmission activity and symptoms seen in schizophrenia. There are many similarities in psychotic symptoms and abnormal neurotransmission activity caused by omega-3 fatty acid deficit and schizophrenia illness.

### Omega-3 supplementation may improve some abnormalities

No one could control over what one inherited, but certain environmental factors could be better managed to minimize the risk of schizophrenia. Intervention with EPA, has been shown to provide beneficial effect on schizophrenia through suppressing the production of inflammatory eicosanoids (by competing with AA for the enzymes, such as cyclooxygenase-2, and cytokines, reducing the susceptibility of neural membranes to oxidative stress, preserve membrane functional integrity, and normal neurotransmission.

Many clinical trials have shown beneficial effects of omega-3 PUFA intervention. In this review, 13 studies which do show clinical efficacy of omega-3 PUFA supplementation on alleviating some symptoms in patients with schizophrenia were included (Table 1). The possible neurophysiological explanations and the potential mechanisms as how omega-3 fatty acids modulate psychophysiological functions and exert their beneficial effects were discussed.

### Possible mechanisms underlying the beneficial action of omega-3 supplementation

The beneficial action of omega-3 supplementation can occur through replenishing the omega-3 content in the brain membrane. Distribution of DHA in the brain is region-dependent. Normally, high concentrations of DHA are found in the frontal cortex and other cortical regions, but low in regions of the midbrain [196–199]. In schizophrenia brain, omega-3 deficit affects most significantly the cortical region, which coincides with the hypoactive dopamine transmission, and negative symptoms. Intervention with omega-3 PUFA, mainly DHA has shown to improve the negative symptoms, suggesting the beneficial effect through replenishing the depleted DHA content in this brain region. Omega-3 PUFAs can reduce the deterioration of brain structure by inhibition of PLA2-induced phospholipid breakdown, restoring and maintaining the brain structures and preserving their function by modulating the membrane phospholipid metabolism, and fluidity, hence, the neurotransmission. Dietary supplementation with omega-3 fatty acids can enhance the incorporation of DHA into brain cells. However, it should be noted that the polymorphism of gene for FABP-7, which transports DHA to brain cells is found to alter the specificity from DHA to LA in some schizophrenia patients. In this incidence, incorporation of DHA into brain cells will be significantly compromised.

The beneficial action of omega-3 supplementation can also occur through gut microbiota. Patients with schizophrenia tend to have poor dietary habits, rich in saturated fats, but low in PUFAs, particularly omega-3 fatty acids [49]. A recent paper has shown a very different gut microbiota in schizophrenia [200]. This difference may modulate brain function through microbiota-gut-brain axis, and affect symptoms [201]. Increasing evidence has shown that dietary supplementation with omega-3 fatty acids affects gut microbiome [202, 203], which in turn, affects neurofunction and mental behaviors [204, 205].

Supplementation with different types of omega-3 fatty acids can result in different efficacy [206]. A recent study by Guo et al. [207] has shown that omega-3 fatty acids, EPA, DPA and DHA were metabolized differently in human. EPA supplementation can raise the levels of EPA in RBC-PL, and EPA and DPA in plasma PL, and CE. DPA supplementation can increase the levels of EPA and DPA in RBC-PL and plasma-PL. However, only DHA supplementation can raise the levels of DHA in plasma PL and CE. Ouellet et al. [208] have shown that EPA and DHA can cross the brain-blood barrier at similar rates, only very low levels of EPA are maintained in the brain due to mechanisms such as active β–oxidation. Thus, the unique role of DHA in neuronal membranes cannot be completely replaced by either EPA or DPA.

### Timing of treatment is important

The onset of full-blown schizophrenic disease occurs typically in late adolescence or early adulthood, during the period of brain maturation, when myelination is continuing [209], dysregulation of PUFAs (including AA and DHA) by the elevated PLA2 activity is also occurring at this early stage [31]. It is critical that intervention carried out before the PUFA deficiency-related neurobiological changes are irreversible [194].

Results from studies by Amminger and colleagues [118–120] have shown that omega-3 fatty acid supplementation to adolescents in an ultra-high risk cohort not only reduced the conversion rate to psychosis in UHR cohort, but also improved both positive and negative symptoms and functions after 12-week intervention, and the beneficial effects continued for a long period (6.7 years). These findings suggest that intervention with omega-3 fatty acids at the prodromal stage can reduce the PLA2 activity and brain degradation [146], while replenish the brain DHA content.

Studies in the first-episode patients received omega-3 fatty acid intervention have also shown improvement in negative symptoms and functions [48, 121–124]. The results indicate that omega-3 supplementation can still exert significant improvement in brain chemistry in newly onset patients. However, omega-3 fatty acid treatment can only improve some symptoms but not functions in chronic patients. A meta-analysis by Chen et al. [192] have concluded that omega-3 supplementation is more effective in reducing severity of psychotic symptoms in young adults or adolescents in the prodromal phase of schizophrenia. Omega-3 fatty acid supplementation can be effective before irreversible neurobiological changes are established [194]. Indeed, a meta-analysis by Chen et al. [195] found that omega-3 PUFAs seemed to be more effective during the early phase of disease (prodrome and first episode), rather than in chronic patients.

### Heterogeneity of patients – omega-3 PUFA baseline and antipsychotic medication

Bentsen et al. [98] have shown two clinically distinct endophenotypes in schizophrenia determined by PUFA levels. Patients with low PUFAs have more negative symptoms than those with high PUFAs [97, 98], and they are more responsive to omega-3 intervention. In these studies, patients all have low PUFA baseline prior to study. Omega-3 fatty acid supplementation raised the blood levels of omega-3 fatty acids.

A recent publication by Cadenhead et al. [210] has shown dietary omega-3 fatty acid intake and plasma RAC were low in individuals with clinical high risk for psychosis as compared to age-matched healthy individuals. Alqarni et al. [211] have also shown that proportions of PUFAs (e.g., EPA, DHA and AA) were significantly lower in the UHR group compared to healthy controls. Amminger et al. [118–120] have shown that omega-3 fatty acid supplementation to adolescents in the UHR cohort not only significantly reduced the rate of transition to psychosis, but also improved the psychotic symptoms.

Antipsychotic medication may improve brain functions and alleviate symptoms (mainly positive and less negative), but it often cause extrapyramidal side effects. The add-on therapy with omega-3 PUFAs may result in a synergistic effect in illness outcomes for UHR adolescents and patients with first-episode schizophrenia. Omega-3 PUFA supplementation can also reduce the antipsychotic dose needed to control the symptoms, increase antipsychotic tolerability, reduce extrapyramidal side effects [119], and improve cognitive performance [212].

These findings support that early detection of PUFA composition and antioxidative status is important to identify the subgroup of patients who may benefit by omega-3 fatty acid supplementation, In light of this, it is recommended that lipid profile, particularly omega-3 fatty acid composition in blood in patients with high risk for psychosis or having first episode be analyzed prior to treatment. Understanding the PUFA status at early stage of the illness can help to identify the population, which can be benefited from the omega-3 fatty acid intervention.

## Conclusion

The current review provides an insight into possible mechanisms underlying the efficacy of omega-3 PUFA in patients with schizophrenia. Omega-3 supplementation is more effective in reducing psychotic symptom severity in young adults or adolescents in the prodromal phase of schizophrenia with low omega-3 baseline. It suggests that patients with predefined lipid levels might benefit from lipid treatments, but more controlled clinical trials are warranted.

## Data Availability

All data generated or analyzed during this study are included in this article.

## Abbreviations

AA: Arachidonic acid (20:4n-6)
ALA: Alpha-linolenic acid (18:3n-3)
BA 10: Brodmann area 10
CNS: Central nervous system
cPLA2: A calcium-dependent AA-specific cytosolic PLA2
CSF: Cerebrospinal fluid
DA: Dopamine (3,4-dihydroxyphenethylamine)
DHA: Docosahexaenoic acid (22:6n-3)
DPA: Docosapentaenoic acid (22:5n-3)
EPA: Eicosapentaenoic acid (20:5n-3)
FABP: Fatty acid binding protein
FAD: Fatty acid desaturase
GM: Grey matter
GPCR: G-protein-coupled receptor
GSH: Glutathione
5-HT: Serotonin (5-hydroxytryptamine)
iPLA2: A calcium-independent DHA-specific PLA2
KYNA: Kynurenic acid
LA: Linoleic acid (18:2n-6)
NMDAR: N-methyl-D-aspartate receptor
PANSS: Positive and negative symptom scale
PFC: Prefrontal cortex
PLA2: Phospholipase A2
PUFA: Polyunsaturated fatty acid
PGE2: Prostaglandin E2
RBC: Red blood cells
RCT: Randomized-controlled trial
sPLA2: A calcium-dependent AA-specific secretory PLA2
SOD: Superoxide dismutase
TAC: Total antioxidant capacity
UHR: Ultra-high risk
VTA: Ventral tegmental area
WM: White matter
